# Transfusion characteristics and hemostatic conditions in octogenarians undergoing emergency surgery for acute aortic dissection: a retrospective study

**DOI:** 10.1186/s40981-020-00358-z

**Published:** 2020-07-09

**Authors:** Tetsuhito Masubuchi, Kenji Yoshitani, Kimito Minami, Chisaki Yokoyama, Akito Tsukinaga, Takahisa Goto, Yoshihiko Ohnishi

**Affiliations:** 1 Department of Anesthesiology, National Cerebral and Cardiovascular Center, 6-1, Kishibeshimmachi, Suita, Osaka, 564-8565 Japan; 2 Department of Anesthesiology, Yokohama City University Hospital, 3-9, Fukuura, Yokohama, Kanagawa 236-0004 Japan

**Keywords:** Octogenarian, Acute aortic dissection, Hemostasis

## Abstract

**Background:**

The number of elderly patients undergoing elective as well as emergent cardiac surgery is increasing. Octogenarian and older patients undergoing surgery for acute type A aortic dissection (AAD) have a significantly higher risk of postoperative mortality than younger patients. Hemostasis is difficult in octogenarians with AAD. However, few studies have investigated perioperative blood transfusion volumes and hemostatic conditions in patients undergoing AAD surgery. We retrospectively investigated whether these factors differed between octogenarians and younger patients with AAD.

**Methods:**

The records of 207 patients who underwent emergency surgery for AAD were reviewed between 2008 and 2014. We compared the total volumes of transfused blood components (red blood cell concentrate, fresh frozen plasma, platelets concentrate, and cryoprecipitate), perioperative blood coagulation test results (prothrombin time-international normalized ratio, activated partial thrombin time, and activated coagulation time), and intensive care unit and hospital stay durations between octogenarians (*n* = 33) and patients < 80 years old (*n* = 170).

**Results:**

A significantly greater volume of red blood cell concentrates was transfused in octogenarians than in patients < 80 years old. Isolated prolonged activated partial thromboplastin time was observed in octogenarian patients. Duration of hospital stays was significantly longer in octogenarians than in patients < 80 years old.

**Conclusions:**

Octogenarians required more red blood cells during surgery for AAD and exhibited isolated APTT prolongation.

## Background

Major surgery is becoming more common in octogenarians as societies worldwide experience rapid aging. The number of elderly patients undergoing elective as well as emergent cardiac surgery is increasing. Acute type A aortic dissection (AAD) is a severe, life-threatening condition that is associated with high hospital mortality (15–30%) [1]. Biancari et al. [2] suggested in a systematic review and meta-analysis that octogenarian and older patients with type A aortic dissection had a significantly higher risk of postoperative mortality than younger patients. Previous studies have shown that operation duration, cardiopulmonary bypass time, and blood transfusion volumes are independent risk factors for mortality in patients with AAD [3, 4]. Clinically, it is often difficult to achieve hemostasis in octogenarians with AAD. However, few studies have investigated perioperative blood transfusion volumes and hemostatic conditions in patients undergoing AAD surgery. In this study, we retrospectively investigated whether octogenarians with AAD differed from younger patients in regard to these factors.

## Methods

We retrospectively reviewed the clinical records and data of patients who underwent surgical treatment for AAD from January 2008 to December 2014. The exclusion criterion was prior thoracic endovascular aortic repair. The study protocol was approved by our institutional review board (17 April 2015, M27-002), and the need for written informed consent was waived due to the retrospective nature of the study. We collected data on the following clinical characteristics: height; weight; levels of hemoglobin and serum creatinine; estimated glomerular filtration rate (eGFR); perioperative blood coagulation tests; intraoperative transfusion volumes of red blood cell concentrate (RCC), fresh frozen plasma (FFP), platelet concentrate (PC), and cryoprecipitate; and durations of intensive care unit (ICU) and hospital stays. Perioperative blood coagulation tests included prothrombin time-international normalized ratio (PT-INR), activated partial thromboplastin time (APTT), serum fibrinogen concentration, and platelet count. All data were collected at four time points: (1) pre-operatively, (2) after protamine administration, (3) at the end of the operation, and (4) at ICU admission. Patients with preoperative hemodialysis were excluded in this study.

### Statistical analysis

All variables are expressed as median (interquartile range). In regard to blood coagulation tests, intragroup differences between patients aged < 80 and ≥ 80 years were examined using a multivariable linear regression model that included a cross-product term between the elapsed time and either age ≥ 80 or < 80 years. This model used the Huber-White method. Statistically significant results indicated that time-series variations of PT, APTT, fibrinogen, and platelet counts differed depending on the age group. To clarify factors affecting total transfusion volumes, backward stepwise multivariable regression analysis was performed. Factors affecting total transfusion volume included age ≥ 80, body weight, eGFR, preoperative hemoglobin concentration, the lowest hemoglobin concentration during cardiopulmonary bypass (CPB), and CPB duration which were reported in previous studies [5–8]. The threshold for significance was *P* < 0.05. Data were analyzed using STATA SE15 and R-3.6.1.

## Results

The records of 207 patients who underwent emergency operations from 2008 to 2014 were reviewed. Of these patients, 33 (16%) were octogenarians and 174 (84%) were aged < 80 years. Baseline data (Table 1) showed that compared to non-octogenarians, octogenarians were shorter, weighed less, and had a lower preoperative hemoglobin concentration and eGFR.

**Table 1:** Patient characteristics and intraoperative data of the group, age < 80 years old and age ≧ 80 years old

	Age≧ 80 yr (*n* = 33)	Age < 80 yr (*n* = 174)	*P* value
Height (cm)	150 (145, 153)	160 (154, 170)	< 0.001
Weight (kg)	48 (42, 50)	60 (51, 70)	< 0.001
Sex (male/female)	6/27	93/81	0.001
Preoperative hemoglobin (g/dL)	10.6 (9.8, 11.7)	12.3 (11, 13.3)	< 0.001
Preoperative platelet (× 10^3^μL)	132 (107, 180)	161 (127, 199)	0.023
Fibrinogen (mg/dL)	239.5 (191.5, 293)	246 (191, 309)	0.60
Preoperative PT-INR	1.03 (0.97, 1.25)	1.03 (0.98, 1.14)	0.50
Preoperative APTT (second)	32.5 (27.5, 39.5)	31 (27.5, 36)	0.34
sCre (mg/dL)	0.84 (0.73, 1.52)	0.87 (.67, 1.09)	0.23
eGFR (ml/min/1.73 m^2^)	51.2 (28.4, 60.8)	62.1 (45.9, 76.4)	< 0.001
Lowest hemoglobin during CPB (g/dL)	7.3 (6.9, 7.6)	7.5 (6.9, 8)	0.22
CPB duration (min)	193 (169, 232)	215.5 (172, 272)	0.18

Data were expressed as median (interquartile range)

*APTT* activated partial thromboplastin time, *CPB* cardiopulmonary bypass, *eGFR* estimated glomerular filtration rate, *PT-INR* prothrombin time-international normalized ratio, *sCre* serum creatinine, *Yr* years old

**Fig. 1:**
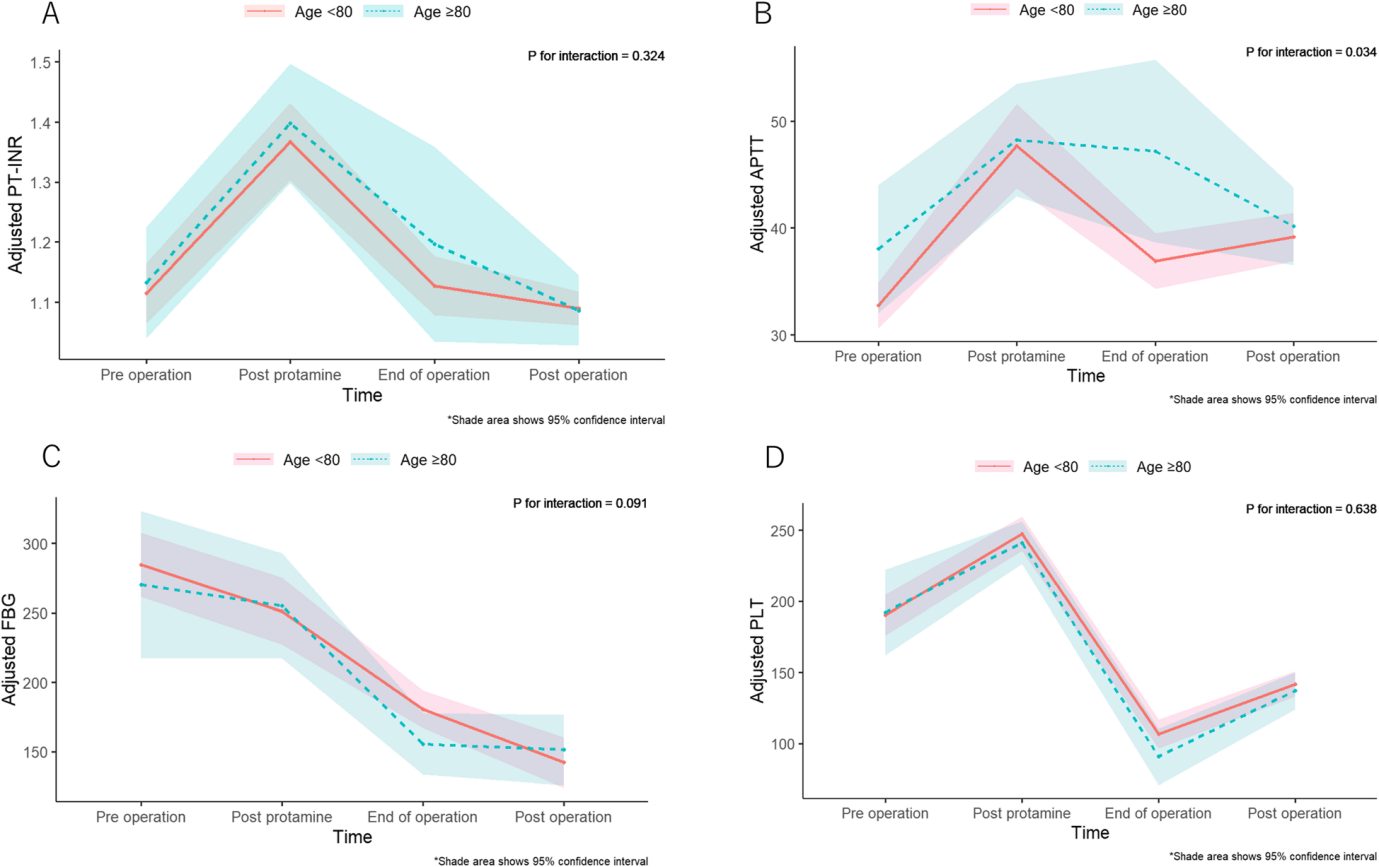
Interaction analysis between elapsed time and **a** prothrombin time-international normalized ratio (PT-INR), **b** activated partial thrombin time (APTT), **c** fibrinogen concentration (FBG), and **d** platelet count (PLT). PT-INR, APTT, FBG, and PLT were adjusted for eGFR, sex, and body weight

Details regarding total intraoperative transfusion volumes are shown in Table 2. A significantly greater volume of RCCs was transfused in octogenarians than in patients aged < 80 years. The results of blood coagulation tests are shown in Fig. 1a–d. Regression models that included an interaction term showed that PT-INR, APTT, fibrinogen, and platelet count were modified depending on age group (Fig. 1a–d; *n* = 118). There was a significant difference in APTT between the age groups (*P* for interaction of APTT = 0.034). Hospital stay durations in octogenarians were significantly longer than those in younger patients (Table 3).

**Table 2:** Total transfusion volumes

	Age ≥ 80 yr	Age < 80 yr	*P* value
RCC (mL)	3886 (1940)	3005 (2010)	0.021
FFP (mL)	2720 (1311)	2743 (1896)	0.94
PC (mL)	906 (382)	799 (394)	0.15
Cryoprecipitate (mL)	95 (129)	70 (97)	0.21
Total transfusion volume (mL)	7607 (3430)	6618 (4048)	0.19

*FFP* fresh frozen plasma, *PC* platelet concentrate, *RCC* red blood cell concentrate, *yr* years old

**Table 3:** Lengths of intensive care unit and hospital stays

	Age ≥ 80 yr (*n* = 33)	Age < 80 yr (*n* = 174)	*P* value
ICU stay (days)	6 (5, 12)	5 (4, 7)	0.055
Hospital stay (days)	36 (26, 52)	26 (21, 36)	0.007

Data were expressed as median (interquartile range); yr: years old.

The results of multivariable regression analysis are shown in Table 4. CPB duration, preoperative hemoglobin concentration, and age ≥ 80 were significantly associated with total transfusion amount.

**Table 4:** Factors affecting total transfusion amounts

	Coefficient	*P* value	95% confidence interval
CPB duration (min)	1.545	< 0.001	13 to 19
Preoperative hemoglobin concentration (g/dL)	− 335	< 0.001	− 499 to − 220
Age ≥ 80 years	768	0.044	1932 to 4962

*CPB* cardiopulmonary bypass

## Discussion

In this study, octogenarian patients undergoing surgery for AAD required a greater RCC transfusion volume. Isolated prolonged APTT was observed in octogenarian patients. Hospital stay durations in octogenarians were significantly longer than in patients aged < 80 years.

As lifespans have grown longer, the number of acute aortic repair operations performed in octogenarian patients has increased. A recent meta-analysis reported that octogenarian and older patients with AAD had a significantly higher risk of postoperative mortality than younger patients [2]. However, surgical intervention for aortic arch disease in octogenarians can yield satisfactory early clinical outcomes and mortality and morbidity rates during hospitalization, and a non-randomized controlled study demonstrated acceptable mid-term survival with adequate daily activity [9]. Therefore, we need to clarify risk factors in this population to avoid unwanted outcomes.

Massive intraoperative bleeding is a serious problem during the aortic repair of acute aortic dissection, since this procedure requires complicated aortic anastomosis and prolonged cardiopulmonary bypass time. Serious bleeding increases the duration of the operation and results in an increased requirement for transfusion of allogeneic blood products.

In this study, age ≥ 80 years was significantly associated with total transfusion amount, a result that was compatible with those of previous reports [1, 10]. Regarding specific blood components, octogenarian patients required a significantly greater volume of RCC than patients aged < 80 years and also demonstrated a significantly lower preoperative hemoglobin concentration. As shown by multivariable regression analysis, preoperative hemoglobin concentration influenced the volume of transfused RCC. Acute aortic dissection consumes red blood cells, coagulation factors, and platelets to facilitate thrombus formation in the pseudo-lumen. The lower preoperative hemoglobin concentration in octogenarians can be explained by their lower body weight. Furthermore, aging leads to unexplained anemia, probably due to pathological processes such as progressive resistance of bone marrow erythroid progenitors to erythropoietin and a chronic, subclinical pro-inflammatory state [11]. Thus, octogenarians are particularly at risk of massive bleeding in acute aortic dissection.

In this study, the APTT was prolonged in octogenarian patients. The APTT reflects the function of the intrinsic pathway, which depends on factors VIII, IX, XI, and XII. A prolonged APTT with normal PT can be caused by one or more of the following: deficiencies in any of the components of the intrinsic pathway, the presence of the lupus anticoagulant or acquired inhibitors of coagulation, systemic anticoagulation (most often with heparin), and von Willebrand disease [12]. After cardiopulmonary bypass, residual heparin and reduced von Willebrand factor (VWF) may cause isolated prolonged APTT. However, many studies have shown that VWF levels are elevated in elderly populations [13–15]. Zindovic and colleagues reported that in acute aortic dissection, VWF activity just before the end of surgery was unchanged compared to preoperative levels [16]. VWF may not be associated with higher levels of APTT just after the administration of protamine.

Regarding residual heparin, APTT is more sensitive than ACT to low-dose unfractionated heparin activity [17]. Low-dose heparin may result in isolated prolonged APTT in octogenarians. Unfractionated heparin is eliminated by binding to macrophages and endothelial cells and by clearance from the bloodstream by the kidneys, the latter of which is a slower process [18]. In this study, eGFR was significantly lower in octogenarians than in patients aged < 80 years. Decreased eGFR may be caused by structural and functional changes that occur with aging in individuals with vascular disease [19] and may be associated with isolated prolonged APTT in octogenarians.

In this study, hospital stay durations were significantly longer in octogenarians with acute aortic dissection. These results are compatible with those of a previous study [20]. Octogenarians may have various complications that can affect the lengths of hospital stays.

There are several limitations to this study. First, it was retrospective in nature, and the patient number was small because eligible patients received emergency surgery. We could not analyze the total volume of intraoperative bleeding due to lack of data and data inaccuracy. As a result, we analyzed total transfusion volume, which may be a surrogate for the total volume of intraoperative bleeding. Second, we did not have a unified protocol for transfusion in the setting of acute aortic dissection. The transfusion volumes of RCC, FFP, and platelets depended on the decisions of attending anesthesiologists, and this may have affected the total transfusion volume. Third, we could not get the data on whether patients had antiplatelet and anticoagulant drugs due to emergency surgery. There might be confounders about preoperative unrecognized hemostatic condition. However, we need to manage AAD without the information of hemostatic condition in most of the case. We believe our finding may help in managing AAD cases.

## Conclusion

During aortic repair of acute aortic dissection, octogenarians demonstrated a greater volume of transfused RCC than patients aged < 80 years and exhibited APTT prolongation.

## Data Availability

The datasets used and analyzed during the current study are available from the corresponding author on reasonable request.

## Abbreviations

AAD: Acute type A aortic dissection
APTT: Activated partial thromboplastin time
eGFR: Estimated glomerular filtration rate
FFP: Fresh frozen plasma
ICU: Intensive care unit
PC: Platelet concentrate
PT-INR: Prothrombin time-international normalized ratio
RCC: Red blood cell concentrate
VWF: von Willebrand factor
